# Postmenopausal Tubular Adenoma of the Breast: A Case Report

**DOI:** 10.7759/cureus.27076

**Published:** 2022-07-20

**Authors:** Brittany Miles, Atefeh Zeinoddini, Arian P Lahiji, Eduardo Eyzaguirre, Quan D Nguyen

**Affiliations:** 1 Medical Education, University of Texas Medical Branch at Galveston, Galveston, USA; 2 Radiology, University of Texas Medical Branch at Galveston, Galveston, USA; 3 Pathology, University of Texas Medical Branch at Galveston, Galveston, USA; 4 Radiology, Baylor College of Medicine, Houston, USA

**Keywords:** lumpectomy, myoepithelial cells, premenopausal women, benign breast lesions, tubular adenoma of the breast

## Abstract

Tubular adenoma (TA) of the breast is a rare, benign proliferative breast lesion that is predominantly composed of closely compacted tubules with an inner layer of epithelial cells and an outer layer of myoepithelial cells. They are regarded as residing on the opposite end of a spectrum of proliferative breast lesions from fibroadenomas, which are predominantly stromal. The majority of TAs are found in premenopausal women and the reason for this demographic predilection is not yet known. It is generally not possible to distinguish between TA and other, higher-risk breast lesions prior to biopsy or resection because the clinical and radiographic findings overlap. In this article, we present the case of a TA in a postmenopausal patient and review the epidemiology, histology, carcinogenic potential, and management of such lesions.

## Introduction

Tubular adenomas (TAs) of the breast are rare, benign lesions that are considered to share a common histogenesis with fibroadenoma and lactating adenoma [1-2]. They comprise approximately 0.13%-1.7% of all benign breast tumors, and only a few cases have been reported in the literature [3-4]. They are pure epithelial neoplasms with homogeneous, tightly packed tubular or acinar components and minimal to no supporting stroma, and usually vary in size from one to five centimeters [5-7]. It has been suggested that a spectrum of histologic appearance exists between the predominantly stromal features of fibroadenoma and the predominantly tubular or acinar epithelial features of TA, with variation in composition and the amounts of tightly packed tubules, dilated ducts, and connective tissue [7-8]. Myoepithelial cell markers [e.g., p63, smooth muscle myosin heavy chain (SMMHC), calponin, S100] can be used to confirm the presence of myoepithelial cells in this benign tumor. In invasive carcinoma, only malignant epithelial cells are present, and myoepithelial cells are lost in the malignant glands. We frequently use p63 and SMMHC in our institution to differentiate benign breast tumors versus breast carcinoma. The differential diagnosis for TA includes tubular carcinoma which lacks myoepithelial markers on histologic analysis. 

One study reported that three factors on ultrasound can assist with the diagnosis of TA when present: macro-lobulation, “tiny branch-like patterns,” and vascularity [2]. That same study found that echogenicity, border, uniformity of echotexture, posterior acoustic enhancement, and lateral wall shadowing were not found to be useful for distinguishing TAs from other breast lesions.

Physical exam and radiographic findings of TA can be indistinguishable from those of fibroadenomas, and surgical excision is generally required for definitive diagnosis [9-10]. TAs larger than five centimeters are designated “Giant” TAs due to the infrequency of achieving that size [3]. Most cases are found in women of childbearing age and postmenopausal discovery is particularly unusual [9, 11]. One study reported that 90% of cases are found in patients younger than 40 years of age, with the upper outer quadrant of the breast the most common location affected [5]. Another study showed that TAs in younger women do not contain calcifications, while those in older patients are associated with microcalcifications suggestive of malignancy, despite the absence of any malignancy or ductal carcinoma in situ (DCIS) discovered after surgical resection [7].

Herein we present a rare case of a postmenopausal female with a TA of the breast.

## Case presentation

**Figure 1:**
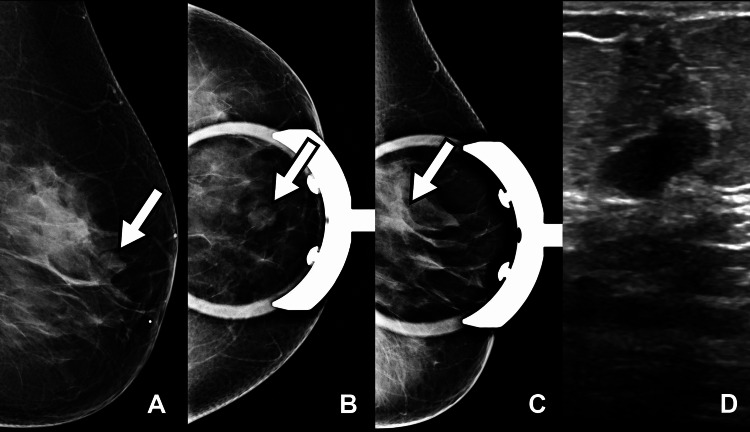
(A-C): Mammogram demonstrates an equal density oval mass with microlobulated margins measuring 11 mm x 11 mm x 5 mm in the upper inner quadrant at 11 o'clock, 2 cm from the nipple. (D): Ultrasound showed an 11 mm x 11 mm x 5 mm solid oval-shaped mass with microlobulated margins and non-parallel orientation in the upper inner quadrant at 11 o'clock, 2 cm from the nipple.

A postmenopausal 48-year-old woman with a premenopausal history of cyclical mastalgia was found on routine screening imaging to have an 11 mm solid mass in the upper inner quadrant of the left breast (11 o’clock position), 2 cm from the nipple. Her family history was negative for breast cancer, and she had no prior history of breast surgery. The suspicious lesion was classified as Breast Imaging Reporting and Data System (BI-RADS) category 4C (50%-94% likelihood of malignancy), and an ultrasound-guided biopsy was recommended (Figure 1).

**Figure 2:**
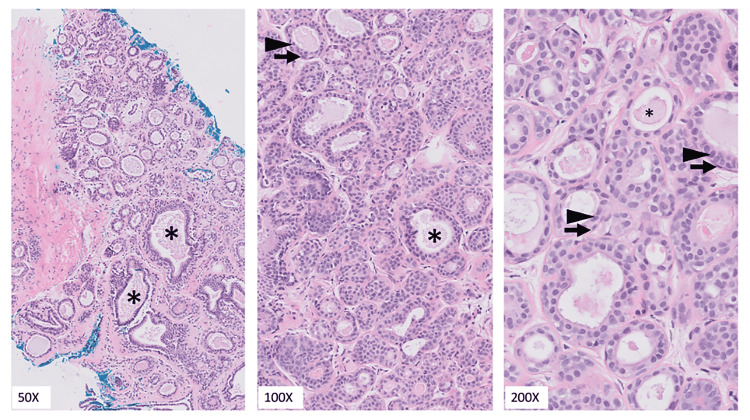
Tubular adenoma. H&E of core needle biopsy shows a lesion with well-defined borders consisting of small, packed tubular structures containing eosinophilic secretions (asterisk) and lined by a basal layer of myoepithelial cells (arrow) and overlying glandular epithelium (arrowhead). There is minimal intertubular fibrous tissue. H&E, hematoxylin and eosin

Physical exam revealed no palpable dominant masses, skin dimpling, nipple retraction, nipple discharge, or peau d’ orange in either breast. No axillary adenopathy was appreciable. A core biopsy was performed, and the diagnosis of a TA was made (Figure 2).

After the biopsy, the patient was presented with the options of surgical resection versus observation with close follow-up. Needle localization and resection was the recommended option due to the possibility that the TA could be contributing to the patient’s breast discomfort.

## Discussion

This case is interesting because a rare, benign breast lesion was discovered in a demographic in whom it is even more rare to find. Because the patient's duration of menopause was relatively brief (one to two years), it also raises the question as to whether the postmenopausal discovery of a TA may happen as a result of a premenopausal lesion escaping detection until after menopause has occurred. 

It is also unknown if the postmenopausal state could promote the involution of TAs because they are usually resected at the time of discovery and not followed expectantly. The observation that calcifications are much more commonly found in postmenopausal TAs could possibly support this theory. Such a question could potentially be answered in the future if a biopsy-proven TA is discovered in a patient who declines or is not a candidate for resection, and the lesion is monitored over time. 

Regarding malignant potential, TAs are considered benign although a small number of reports have found them to exist in close proximity to malignancy, or have suggested the possibility of malignant transformation [12-13]. There has been at least one report of DCIS developing within a TA, and another stating that transformation from TA to fibroadenoma had likely occurred [12-13]. Cases have also been seen in which a TA and ductal carcinoma are in direct contact, but with a clearly defined border between the lesions, attributed to a desmoplastic reaction [14].

Even if a solitary TA is suspected based on radiologic appearance, a biopsy is still recommended in most cases to exclude the possibility of malignancy [2]. If TA is confirmed by biopsy and the patient does not desire surgery, surgery may be avoided although close follow-up has been recommended as a precaution [2].

Tubular adenoma and fibroadenoma appear to share similar histogenesis, but they differ slightly with regard to treatment. Simple, asymptomatic fibroadenomas do not require resection. If a fibroadenoma increases in size or becomes symptomatic, then resection is recommended. For TA, surgical excision is generally performed in order to exclude the presence of synchronous malignancy. If none is found, then excision is the only intervention required and there is no role for additional treatment. Any patient who does not undergo resection should be monitored for any changes over time. 

## Conclusions

Tubular adenomas of the breast are rare, benign lesions that can be difficult to distinguish from other benign or malignant breast masses without histologic confirmation because the radiographic and physical exam findings do not facilitate definitive diagnosis. For these reasons, and to exclude a concurrent malignancy, TAs are usually resected upon discovery. Based on the demographics of diagnosed patients, it is reasonable to suspect that a premenopausal state is more conducive to the development of TAs than a postmenopausal one. Hopefully, the mystery of TAs in the breast will become clearer as additional cases are discovered over time.
